# 撤稿声明

**DOI:** 10.3779/j.issn.1009-3419.2023.102.21

**Published:** 2023-06-20

我刊2017年第20卷第3期刊登的题名为《阻断EGFR影响肺鳞癌肿瘤微环境Treg细胞及IL-1A表达》（doi: 10.3779/j.issn.1009-3419.2017.03.01）一文因涉嫌剽窃，现编辑部做撤稿处理，特此声明。

编辑部将进一步加强期刊出版学术规范问题审查，避免此类事件再次发生。

中国肺癌杂志编辑部

2023年6月

The article titled “Altered Treg and IL-1A Expression in the Immune Microenvironment of Lung Squamous-cell Cancer after EGFR Blockade” (doi: 10.3779/j.issn.1009-3419.2017.03.01) plagiarized the previously published article. Now Editorial Office of Chinese Journal of Lung Cancer has decided to retract it.

The editorial office will strengthen checking procedures for the academic publishing specification to avoid the recurrence of such events in future.

Editorial Office of Chinese Journal of Lung Cancer

June, 2023

